# Defence against Black Hole and Selective Forwarding Attacks for Medical WSNs in the IoT †

**DOI:** 10.3390/s16010118

**Published:** 2016-01-19

**Authors:** Avijit Mathur, Thomas Newe, Muzaffar Rao

**Affiliations:** Department of Electronic and Communication, University of Limerick, Limerick V94 T9PX, Ireland; Thomas.Newe@ul.ie (T.N.); Muhammad.Rao@ul.ie (M.R.)

**Keywords:** routing attacks, black hole, selective forwarding, sensor networks, medical WSN, IoT-Internet of Things

## Abstract

Wireless sensor networks (WSNs) are being used to facilitate monitoring of patients in hospital and home environments. These systems consist of a variety of different components/sensors and many processes like clustering, routing, security, and self-organization. Routing is necessary for medical-based WSNs because it allows remote data delivery and it facilitates network scalability in large hospitals. However, routing entails several problems, mainly due to the open nature of wireless networks, and these need to be addressed. This paper looks at two of the problems that arise due to wireless routing between the nodes and access points of a medical WSN (for IoT use): black hole and selective forwarding (SF) attacks. A solution to the former can readily be provided through the use of cryptographic hashes, while the latter makes use of a neighbourhood watch and threshold-based analysis to detect and correct SF attacks. The scheme proposed here is capable of detecting a selective forwarding attack with over 96% accuracy and successfully identifying the malicious node with 83% accuracy.

## 1. Introduction

For several years wireless sensor networks have been gaining popularity in the healthcare industry among other domains. Each domain may have a different purpose and different reliability, dependability, and availability specifications. For example, a healthcare monitoring system [1,2] may need higher reliability than other systems. This is because the main aim of a patient monitoring system is data delivery. If this objective is nullified in any fashion then the reliability of the system decreases. In-order to protect the system from these types of downfalls we must secure it against attacks that can render the system useless despite the implementation of encryption and authentication mechanisms. These attacks are collectively known as Denial of Service (DoS) attacks [3], some of which are explained in detail in Section 5. Before going into the details of routing and its security let us begin by examining WSNs, their components and usage in the medical WSN industry.

**Figure 1:**
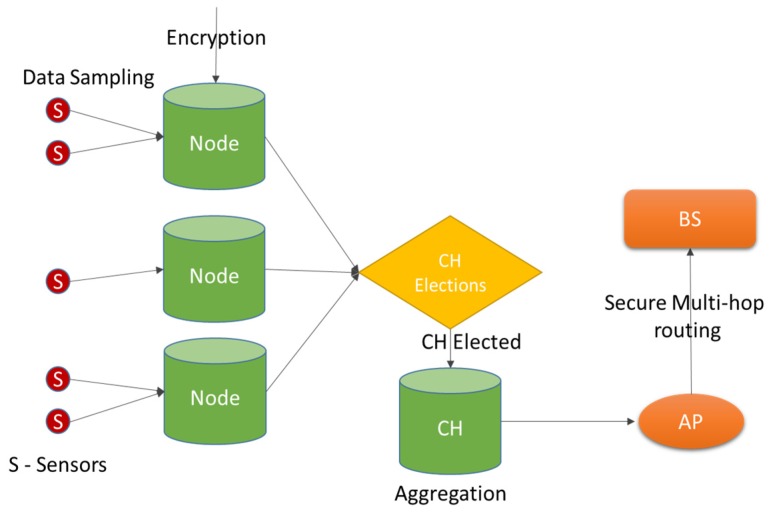
System model.

Figure 1 illustrates our system model for a medical WSN. Here, the system comprises of different components like sensors (S), nodes, clusters of nodes, cluster head (CH), access points (AP), and base stations (BS). The sensors sample raw data and send the values to the nodes which may convert data and encrypt it for security. Following this the nodes elect a cluster head and its backup *i.e.*, a shadow (SH). The CH aggregates the encrypted data and sends it to the nearest AP which in turn sends it to the BS through a secure multi-hop routing process. To facilitate this the system defines a routing protocol that is capable of carrying the data from source to destination by using either static routes or dynamic routes. The former is apt for static networks while the latter is good for mobile networks, namely medical wireless sensor networks (MWSNs).

Now when it comes to routing in a MWSN some important factors should be noted. First, the network must be reliable enough so that data always reaches the BS. This is achieved by using a mesh routing protocol as it provides reliability through path redundancy. Second, the protocol must be secure against common routing attacks. In our system we will be focussing on two common and high-impact DoS attacks; namely, black hole and selective forwarding. These are further explained in Section 5.

Finally, the advantages of using WSNs in IoT healthcare environments are: (1) they allow patients to be mobile, *i.e.*, a patient can get their vitals monitored whether they are at home, work, or at play; (2) it enables the doctor to get real-time values of a patient’s vitals. It allows medical staff to obtain data related to a “surprise” event that could not be foreseen in relation to a patient’s well being. Generally, these “surprise” events are difficult to monitor/predict and so medical staff often do not get valid data for the event; and (3) finally, the WSN system reduces the workload of the duty staff [3] due to its constant monitoring and data logging capabilities.

The remainder of the paper is structured as follows: in Section 2, work related to detection and correction of the above mentioned attacks is discussed. In Section 3, the system overview, its components, and architecture are outlined. In Section 4, the cluster elections are introduced in-order to show the aggregation of data. In Section 5, the problem that denial of service attacks present is outlined. Here a scheme for preventing the black hole attack and a scheme for detection and correction of SF attacks are presented. Section 6 reviews the modifications made to the protocols of the Contiki OS to achieve a defense against black hole and SF attacks. Section 7 presents the measured results for an implementation of the modified and original protocols on the Tmote Sky [4] and OpenMote [5] platforms. Finally, the conclusion summarizes the changes made and their success at detecting black hole and SF attacks.

## 2. Related Work

Medical-based WSN systems are susceptible to various attacks that can harm the system and/or the people associated with it. Hence, research into security and defense mechanisms has been going on for quite some time. Some of the security work for medical WSN include [6], where the authors have formulated a secure data transmission protocol, and discussed issues relating to integration of security in medical WSN. In [7] the authors provide a lightweight and secure system for medical monitoring. Their implementation uses hardware with limited resources and provides secure data transmission with access control. Other healthcare-targeted issues which are security related in WSN can be found in [8,9,10].

Considering the security of WSNs we will begin by describing work related to black hole and selective-forwarding DoS attacks. In [11] , the authors propose two different solutions to the problem of black holes in wireless sensor networks (WSNs). The first solution uses the multi-path property of redundant networks. The sender node waits for multiple response (RREP) packets to arrive and then decides which path is safer by eliminating the unsuitable path. The second solution requires sequence numbers. This is to keep track of the last packet “sent to” and “received from” every node.

The problem with the first solution is that it requires multiple shared hops between source and destination nodes. This hopping can incur time delays if there are not enough paths available in the network. On the other hand, the second solution incurs lower time delay but has no way of re-sequencing the packets received at the receiver. This may lead to garbled transmissions. Moreover, for both solutions the authors did not use any attacker nodes and they verified packets by verifying the routes only, which could result in false positives. Finally, they did not address the problem associated with a collaborative black hole attack, which involves the collaboration between multiple malicious nodes to bring down a network, as is the usual scenario in real world implementations.

In [12], the authors used threshold values and neighboring promiscuous nodes to detect the black hole node in a mobile *ad hoc* network (MANET). The issue with this solution is that each node holds extra routing tables, thus leading to storage overhead on the nodes.

In [13], the authors describe their scheme as being capable of defending against single and collaborative black hole attacks for MANETs. They state that it is successful even when the node is idle. However, the scheme has separate mechanisms for idle and communicating nodes. When compared to the Ad hoc On-Demand Distance Vector (AODV) protocol [14] the end to end delay increases by 100 ms to 300 ms for different numbers of attacker nodes *i.e.*, 1, 4, and 5. The scheme also does not consider the grey-hole (selective forwarding) attacks, where the malicious nodes drop packets selectively to avoid suspicion.

In [15], the authors simulate a multipath routing mechanism on the Omnet++ simulator for WSNs. In this mechanism they improve the reliability of the network by modifying the routing protocol. Here a node overhears the transmission of neighbor nodes. If there is dropping of packets the node chooses an alternative path. This results in a reduced packet drop ratio, however, there is no mechanism proposed to isolate the malicious node causing the problem.

In [16], the authors combine the geographic routing [17] and watermark-based schemes [18] to achieve high efficiency against selective forwarding attacks in WSNs. The former allows the system to select a secure routing path while the latter is capable of finding and isolating the malicious nodes one at a time. However, the malicious node detection mechanism consumes higher energy than normal mode and there are transport delays due to the watermark extraction process. Moreover, it may be possible that a node becomes compromised after the process of selecting a secure route, thus falsifying the normal packet loss ratio. This could lead to undetected selective forwarding attacks in the later stages of the system.

The scheme presented in [19] is suitable for tree-based protocols. The authors use watchdog nodes to monitor communication links between nodes in WSNs. The watchdogs count packets in a given time window and the cluster head collects data from these watch dogs and then analyzes it using probability theory. However, it is difficult to scale this architecture to large networks as the required number of watchdog nodes will be higher and the traffic will be denser.

The proposal in [20] adds security to multipath routing of WSNs. This is done by using MD5 authentication and encryption for confirming identities and securing multipath, respectively. However, the use of encryption in routing can result in unnecessary overhead even before data transmission. Moreover, the authors do not provide any simulations or results to support the underlying mechanisms and algorithms.

Most of the schemes mentioned above either consume considerably higher energy than the original system or they are unable to isolate the malicious node. The system proposed here achieves a minimal gap ranging 141–293 μW between the power consumption of the original system and the secure system (see Section 6). It also provides the ability to isolate the malicious node(s) and form a new path free of any malicious node. Moreover, the related works mentioned above prove their methods using simulations only. However, this work here provides both simulation results (Tmote Sky platform) and hardware test bed results using the OpenMote platform. This allows real-world test bed results to be obtained and verified in conjunction with simulation based measurements.

## 3. System Overview

In the medical-based WSN system used in this work there are components, processes, protocols, and objective(s):

1. Components refer to sensors, nodes, access points, base station(s), databases, and servers. Out of these components some of the important ones that will be covered in this paper are nodes, access points, and base stations.
2. Processes are the steps taken to achieve a particular objective. These may be acquiring patient data, sending control signals, network maintenance, route setup, and so on. This work will focus on route setup with basic maintenance to facilitate a self-healing network.
3. Protocols are the set of rules followed by the network communications. This work focuses on the lower-layers of the Contiki operating system (OS), namely the radio duty cycling layer and the network layer.
4. Objective refers to the aim of the system to help reduce the burden on the duty staff; increase patient mobility and increase system reliability.

**Figure 2:**
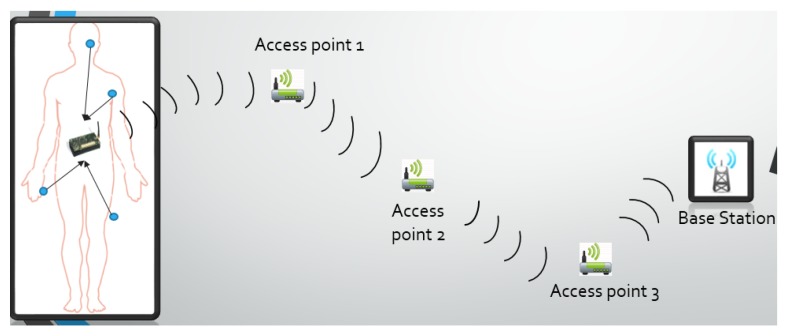
System overview.

The system derived from these factors can be seen in Figure 2. Here the first noticeable area is the patient. This patient has nodes with sensors attached to them. These sensors can perform heterogeneous tasks like measuring temperature, blood saturation levels, electrocardiogram (ECG), electroencephalogram (EEG), and gait analysis. The sensors send sample data to their respective nodes. These nodes are then responsible for encrypting the data using the standard AES-256 cipher from National Institute of Standards and Technology (NIST). One of the nodes then aggregates and transmits encrypted data to the nearest access point which routes the data to the base station (BS). Finally, the BS transmits its data through the Internet for storage and processing on more powerful devices which may then be accessed by the hospital staff.

To achieve the above mentioned objectives the Contiki OS is used. Contiki is used as it provides a number of advantages over other operating systems [21,22], one of them being that different radio sleep times can be enforced by different medium access (MAC) layers and radio duty cycling (RDC) layers. For example, nullrdc provides no duty cycling at all, while ContikiMAC allows nodes to sleep 99% of the time [23]. This implies there is no need to implement a separate sleep-controlling interface as implemented in [24].

Now, each of the above mentioned components and processes are described in the following sections. First, cluster head elections for nodes are discussed in Section 3.1, then routing is discussed in Section 3.2.

### 3.1. Cluster Elections

As mentioned above, the primary job of the sensors attached to the patient’s body is to sample raw data and send it to the node. However, this data must reach its final destination (BS) safely. To facilitate these requirements some measures are taken.

First, it is not viable for all nodes to transmit data individually to the nearest access point (AP). This is because packet collisions and/or battery depletion leads to data loss. One solution involves the sensor nodes electing a cluster head (CH) amongst themselves using a weight calculation procedure that uses:

- Link quality indicator (LQI).
- Time previously served as CH.
- Time spent by the node in each of its power states (t_ps_).

Considerations involving the first two points above are well known and, hence, we shall not dwell on them. The third point *i.e.*, the inclusion of t_ps_, the time spent in the power states is directly proportional to the energy consumption (E_ps_) of the node Equation (1):

Eps = (V_cc_ × I_c_ × t_ps_) (1)

In addition, the energy consumed is directly proportional to the amount of battery consumed (C_c_). The usage of the battery remaining parameter (C_r_ = C_total_ − C_c_) has been disregarded because:

- Larger battery nodes are favoured due to the usage of C_r_ in the linear weight calculation for the system in [24]. However, in our method CH election depends on C_c_ that is independent of C_r_.
- This is advantageous as all nodes have a fair chance of becoming a CH. The method is not biased towards a certain number of nodes having a larger power source. However, the disadvantage is that nodes with smaller batteries may shut down earlier than nodes with larger batteries.

The authors in [24] have also provided a non-linear weight calculation; however, it can lead to a stagnant CH election process. This happens when the last node has used the first 20% of its battery charge, thus inhibiting the re-election of CHs and leading to excessive energy consumption. However, when using our t_ps_ method the system may never reach this situation because it does not consider the “percentage of battery” used but the “amount of battery used irrespective of the battery’s total charge”.

Following the successful election of the CH the nodes transmit their data to the CH. It then aggregates the encrypted data and transmits it to the nearest AP. This AP in turn routes the data to the BS using a modified version of mesh routing which is described in Section 3.2 and Section 4.

### 3.2. Routing

**Figure 3:**
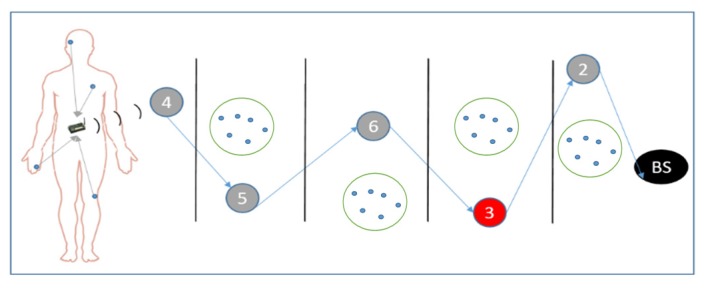
Network model.

Before proceeding into the realms of routing lets look at the network model of our system, Figure 3. This model is based upon the sink-collection model presented in [25]. Here, each access point (AP) collects data from its cluster of sensors (through the CH) and periodically routes this data towards the base station. This period is decided by the BS and is received by the access points during the routing phase, see Section 4.2.

Considering the routing, the network uses an on-demand routing protocol similar to the AODV protocol [14]. This protocol assists the network in sending packets from a source node (close to a patient) to the base station (accessibly by hospital staff) on a mesh network. This mesh network is incorporated so as to enhance reliability through node redundancy [26].

This routing protocol is dynamic in nature, making the system self-organising and self-healing in order to facilitate the movement of patients. It is self-organising as it uses an on-demand approach for setting up the route, and is self-healing because it checks for breaks in the routing path due to the movement of patients or failure of nodes. The protocol also facilitates the addition of a new node when the latter sends a beacon into the cluster. This allows the access point (AP) of that cluster to re-initiate CH elections. If a CH or shadow (SH) is removed from the cluster then the AP identifies this through the absence of the periodic beacon. If the AP confirms that CH or SH is removed it then re-initiates the CH elections. However, if a normal node is removed from the cluster the CH and SH will note its absent periodic interval. They will then remove the entry of this normal node from their lists. This allows easy addition and/or removal of sensor nodes to/from a patient’s body.

## 4. Problem Statement

Up to now we have talked about data passing through nodes, cluster heads, and access point(s), but what happens to the encrypted data during the routing phase? The system must be capable of routing this data securely through the network. This is because in a medical-based WSN the most important requirement is the safe delivery of patient data at the base station *i.e.*, the medical staff computer. If this information were to be mishandled or altered in any way, then the system and the patient may be in jeopardy. Hence, it is a requirement that the system must be protected against attacks capable of the above consequences. These attacks are a class of attack known as Denial of Service (DoS) attacks. Interested readers may refer to the overview on DoS attacks, methodologies, and countermeasures in [27]. DoS attacks encompass a wide array of specialized or generic attacks that may be host-based or network-based. In this work the focus is on two DoS attacks *i.e.*, black hole and selective forwarding (SF).

Black hole and selective forwarding are types of DoS attacks that may harm the medical WSN more than other systems. This is because the aim of a medical WSN system is data delivery; and these attacks render the system useless by denying the delivery of data/control signals. Hence, due to the impact of these attacks on data delivery systems we shall explain them in detail, and present counter-measures using the model of a medical-based WSN system.

### 4.1. Problem: Black Hole Attack

In a medical WSN system, using the routing protocol mentioned in Section 3.2, an attacker can make use of the dynamicity of the protocol to listen to the request (RREQ) packets and use it to their own advantage. This is achieved by replying with a forged RREP packet that advertises the shortest path to the destination. Thus forming a connection between the source node and the malicious node. Following this, the fate of every packet on that route is in the hands of the malicious node.

The above mentioned process is known as a black hole attack [28] and is a cause of concern for our medical-based system because:

1. The medical monitoring system will be at the mercy of the malicious node(s), thus sabotaging the main goal of the system that is patient data delivery.
2. Physical security of the patients may be compromised. This is because patient’s vitals can be read and/or altered by the malicious node(s).
3. Workload of the medical staff may be increased, as the malicious node may trigger false alarms.

**Figure 4:**
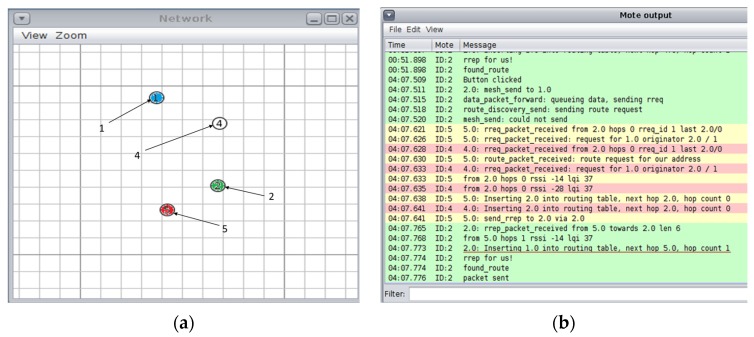
Black hole attack simulation on Cooja simulator (Contiki): (**a**) nodes layout. Source node green “2”, destination is blue “1”, and malicious node is red “5”; and (**b**) Mote output: node “2” sending data packets to node “1” via node “5”, but these packets never reach their destination.

In order to get a better perspective of the black hole attack a simulation was ran using the Contiki OS (the cooja simulator [29] was used) that consists of four nodes: one base station, two normal nodes and one malicious node (Node ID: 5). This simulation is outlined in Figure 4a. Here, node ID “5” denies service to the network by forging a RREP packet. The malicious node “5” then sends a forged RREP to node “2” (see underlined text), Figure 4b. Following this, node “2” sends data packets to node “1” via node “5”. However, these packets never reach their destination because the actual path should be from “2” to “4” to “1”.

From the discussion above it is clear that the black hole attack is capable of making the medical monitoring system useless. Therefore a defence mechanism for this attack is necessary. This mechanism is outlined in the Section 4.2.

### 4.2. Proposed Solution for Black Hole

Here the mechanism used to defend the system against a black hole attack is discussed. In order to proceed further a necessary assumption is that the base station (BS) is trustworthy. This is a basic requirement to facilitate a decision whether a node is malicious or normal as it rules out BS involvement in the attack.

**Figure 5:**
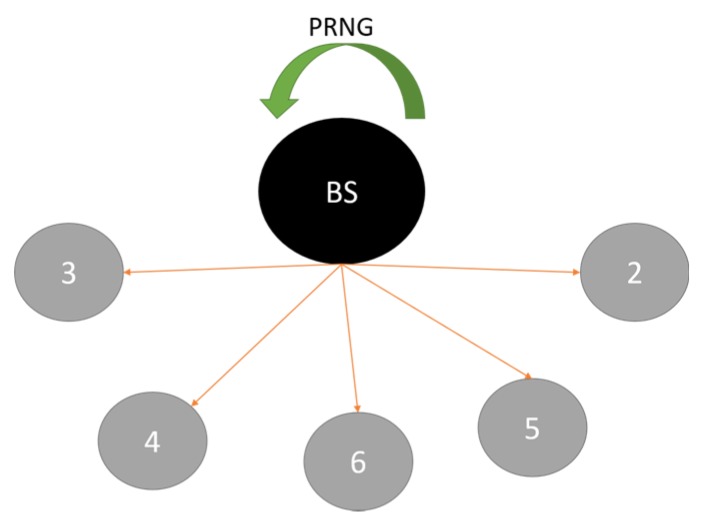
Pre-Deployment process: The access points receiving their respective unique random numbers from the BS.

The method to determine if a node is malicious or not is divided into two phases *i.e.*, pre-deployment phase and routing phase:

Pre-deployment phase: Figure 5 illustrates the initial phase of the system. During this phase every access point (AP) is in-range with its base station (BS). This is done to facilitate the distribution of unique random numbers from the BS to APs. The numbers are generated using Contiki’s pseudo-random number generator and a custom seed generator. Where the latter uses the unique node ID as an initial seed to generate a final seed [24]. The usage of this random number is explained in the routing phase below. After the generation of numbers, the BS distributes them using unicast messages. Here, it is assumed that this is a controlled delivery and that there is no malicious node listening to these messages. Following this the access points are placed in their respective positions of the network similar to the one presented in Figure 3 so that the routing phase can begin.

After the completion of this phase if there is a requirement for AP addition it can only be done for the APs that are not acting as source nodes (data senders). The mechanism necessary for re-authenticating the source APs is proposed for future work and is not covered in this paper.

**Figure 6:**
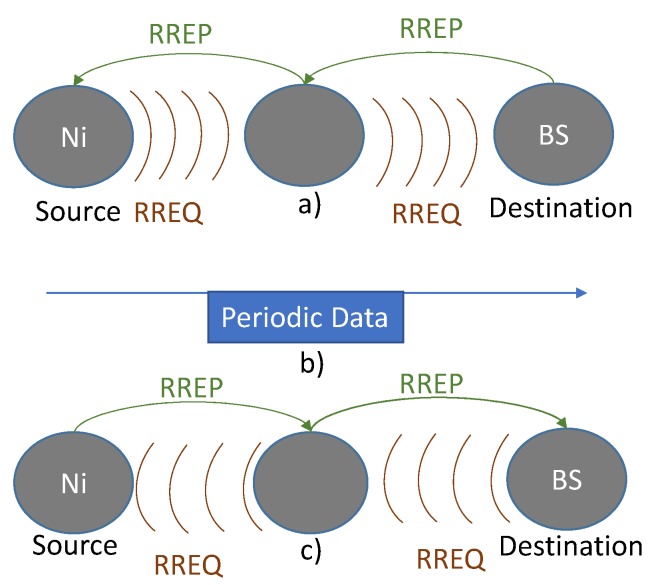
Routing Phase: (**a**) AODV protocol; (**b**) data direction; and (**c**) modification for our system.

Routing phase: The routing protocol is modified to suit the needs of our medical WSN system. The first modification is the reversal of the senders of RREQ and RREP packets , *i.e.*, the destination node (BS) sends RREQ packet and the source node (Ni) replies with a RREP packet, Figure 6. This modification has the following advantages:

1. Reduced network traffic compared to original AODV protocol because an attacker cannot flood the network with bogus RREQ packets, thus reducing the chances of a DoS attack.
2. The network is controlled by the base station which is capable of setting the data intervals for the access points, thus an attacker is incapable of changing the data interval, thereby reducing further possibility of a DoS attack.

**Figure 7:**
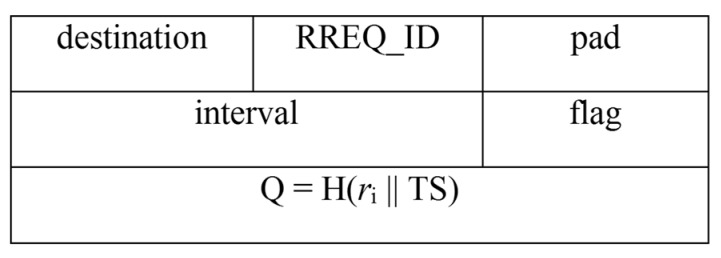
RREQ Packet.

Now, the routing process starts when the BS requires data, this may be the case when an external request (by the staff) or an automatic periodic request (by the BS) is made. When the BS recognises that it needs patient data, it sends a request to the source node Ni using the RREQ packet. The structure of which is shown in Figure 7.

As shown above the RREQ packet consists of a field named Q, where Q = H(*r*_i_ || TS). Here, H refers to the SHA2 hash algorithm, *r*_i_ is the random number for the node, and TS is the timestamp. The reason for this modification is that it provides source authentication and prevents replay attacks. The former implies that the node N*_i_* is assured that the RREQ packet originated at the BS only, while the latter implies that an adversary cannot replay old messages to facilitate penetrating the routing path. The step is shown as follows:

1. The BS sends (RREQ || Q || TS) to N*_i_*
2. When the source N_i_ receives this route request message it first checks for a replay attack by verifying the timestamp:
  a. N*_i_* calculates TS_d_ = TS_current_ − TS_received_.
  b. If TS_d_ > Threshold (set by analysing simulation data) then reject the request and wait for another one.
  c. Else calculate the hash Q’ in the same way Q was calculated.
3. Once the hash Q’ is verified by source N_i_ it then:
  a. Sets the interval that is embedded in the request packet, Figure 7.
  b. Sends back the reply packet (RREP) to the BS, Figure 6c.
  c. Finally, starts sending periodic data to the BS, Figure 6b.

From the above steps it is safe to say that a RREQ packet cannot be forged because of the inclusion of the hash. Therefore, a malicious node cannot request data from the normal nodes in its pursuit of a black hole attack.

In addition, there is no inclusion of a hash in the RREP packet. This is because it would be fruitless for a malicious node to forge a RREP packet and start sending data to the BS. Since it is understood that the objective of a malicious node is to deny data from reaching the BS. The advantage of this exclusion is that it saves unnecessary processing at the nodes’ side, thereby consuming less energy.

### 4.3. Problem: Selective Forwarding

In this section the selective forwarding (SF) attack is described. The selective forwarding attack is a specialised version of the previously mentioned black hole attack. In this attack [30] an attacker captures and compromises single or multiple nodes in order to launch the attack. The attack is known as single selective forwarding or collaborative selective forwarding, respectively.

When a malicious node is performing an SF attack it may drop only a certain number of packets. This helps the node in maintaining its cover by not arousing suspicion because wireless networks are lossy by nature. Therefore, due to packet losses from interference and from the SF attack it can be difficult to distinguish the reason behind packet loss in a WSN. Now, the reason these packet losses may be harmful for a medical WSN are as follows:

1. Incomplete information, reaching the BS, can be more dangerous than no information. This is true because in medical terms one may not see the complete picture without all the data.
2. Altered patient information may be reaching the BS. This could result in incorrect treatment of the patient, thus physically harming the patient.

In order to safeguard the system against this SF attack a mechanism has been devised in this work. This mechanism will help protect the system against single and collaborative SF attacks. The following section describes this mechanism in detail.

### 4.4. Proposed Solution for Selective Forwarding

In this section the mechanism for defending against selective forwarding attacks is detailed. Here it should be noted that the BS and the data sending source node (N*_i_*) are trusted. These entities are trusted because the RREQ packet cannot be forged according to the hash-based scheme presented in Section 4.2.

The details of this mechanism is divided into five sections: neighbour monitoring, attack detection, control packet collection, analysis and new path.

**Figure 8:**
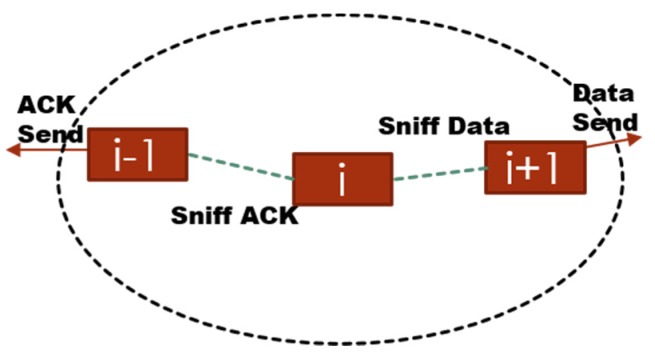
Neighbour monitoring process.

Neighbour Monitoring: Since the routing protocol is on-demand that means that every node has knowledge about its immediate neighbour only. Thus, in order for the system to detect the malicious node(s) the BS needs to have information on all of the network nodes. To achieve this objective every node (*i*) is promiscuous *i.e.*, they monitor data sent by their next node (*i +* 1) and acknowledgement sent by their previous node (*i* − 1), Figure 8.

This promiscuity and monitoring process is achieved by modifying the radio duty cycling (RDC) layer of the Contiki OS, see Section 5. The layer monitors packets only if a particular node falls on the route responsible for sending data from source to BS. This is required to avoid other nodes from monitoring packets unnecessarily.

In addition, regarding the monitoring process, the BS monitors every ten data packets (within a certain time). It counts whether packets were dropped or sent or received. The packets thus generated are called control packets (CP). These are collected at the BS for analysis after the attack detection process is completed.

**Figure 9:**
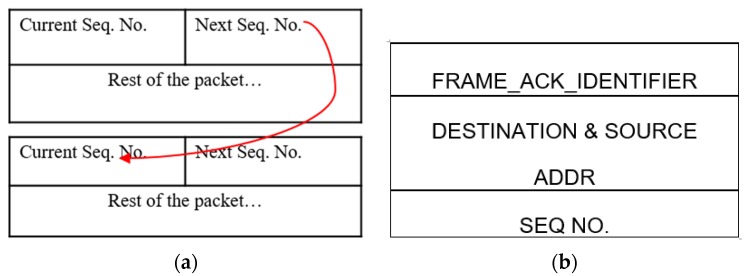
Packets: (**a**) data Packet; and (**b**) ACK packet.

Attack Detection: In our system, an SF attack is first detected at the base station. The reason for this centralized approach is that it does not give unnecessary authority to access points or other nodes. In this process a unique sequence number embedded in the periodic data packet is used. Figure 9a shows data packets containing current sequence number and the next sequence number. Where the latter refers to the sequence number of the next data packet. The usage of these sequence numbers is advantages because:

1. They introduce randomness in the sequencing (through a PRNG-Pseudo Random Number Generator).
2. Allow the BS to keep count of the packets.
3. Can prevent replay attack if the attacker does not know or have access to the sequencing algorithm.

The BS will acquire the first sequence number during the route setup as the source sends the first random Seq. No. attached in the RREP packet. This allows the BS to know the sequence number of the first “data” packet. Following this, every data packet carries the Seq. No. of the next packet. If the Seq. Nos. do not match at the BS, the BS increments a count for the number of packets dropped. If the packet-drop count increases beyond a certain threshold, then the BS deduces that the system is under a selective forwarding attack. Here, the threshold adjusts dynamically over time depending upon the number of packets received. This process is supported by the assumption that the packets do not arrive out-of-sequence. Following this phase, the BS requests control packets from the network nodes.

**Figure 10:**
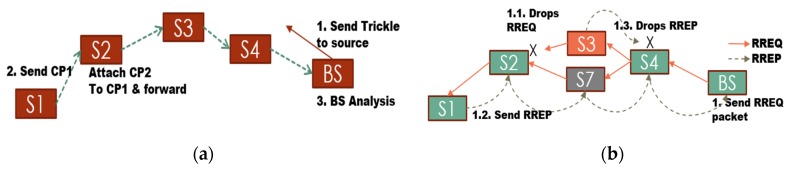
Selective forwarding: (**a**) detection and correction process; and (**b**) new path formation.

Control Packet Collection: Figure 10 illustrates the collection of these control packets at the BS. The source unicasts the first CP (CP1) to its next hop. If the next hop does not forward the message (within a certain time), then the source floods the CP1 using netflood. When another node (on route path) receives this CP1, it appends its own CP to CP1 and forwards it. In this manner the number of transmissions are reduced drastically compared to the scenario when nodes were simply flooding the individual CPs. This mechanism is explained in detail in Section 4.5.

Finally, when the CPs are received at the BS, the BS begins analysing them to determine the ID of the malicious node(s).

Analysis: The BS analyses CPs by monitoring the number of transmissions sent and received by each node. It then tallies these numbers with the number of data packets received. Finally, using a threshold (decided by running the system repeatedly), the BS is able to identify the malicious nodes on the routing path between the BS and source. This process is explained in detail in Section 4.6.

New path: Moving on, the BS sends the information about malicious nodes into the network using the trickle protocol of Contiki (reliable single-source flooding). Upon receiving this message every node in the network drops any packet (RREQ/RREP or data) “originating from” or “forwarded by” the malicious node(s). Thus, the new path request (RREQ) by the BS results in a routing path that is free from any malicious node(s), Figure 10b.

To illustrate this process, video components are available and accompany the electronic version of this manuscript. Please refer to the Supplementry material section at the end of this manuscript.

In the following sections details of control packet collection and packet analysis at the BS are presented.

### 4.5. Packet Gathering

Packet gathering refers to the collection of required data (patient information) at the BS. The mechanism used for gathering data may be flawed with inefficient energy consumption and unbalanced load distribution. For example, energy consumption may be increased if a node transmits data directly to the BS, thereby avoiding its next-hop malicious node. Therefore, in this section a local fix is discussed *i.e.*, without long range transmission capability to the BS, for a scenario where control packets (CPs) may be dropped during data gathering.

**Figure 11:**
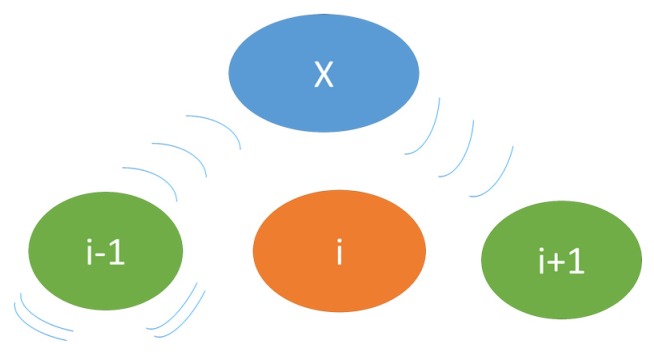
Fix for scenario when malicious node drops CPs.

Figure 11 illustrates the mechanism required to fix the above mentioned problem. The roles, in this case, are distributed to two layers *i.e.*, the application layer and the RDC layer. The application layer re-organises the network if a node (say node *i*) is dead or dropping packets. This layer uses a timeout interval and invokes the RDC layer to monitor the node’s neighbour. If within this specified time a packet is not sent by the neighbour node then the current node *i* − 1 floods the network in a controlled fashion.

This flooding does not include the nodes that are the previous hop of the current node, thereby reducing traffic to some extent. When the node *i* + 1 receives the netflood it cancels the rebroadcast and forwards the desired packet onto its next hop, thus resuming the network mesh protocol and avoiding DoS.

Looking at previous work related to gathering mechanisms, the authors of [31] present a mechanism called AntChain. In this mechanism every node aggregates data from its previous hop, attaches its own data and forwards it onto the next hop. If a node does not receive data then in the next and following round it will assume that the neighbour is dead and transmit data directly to the BS. This means that more energy will be consumed due to a long-range single-hop transmission. However, in the method presented here the adjustment is purely local. The system sends a local broadcast (netflood) instead of a longer range unicast (to BS), thus the network will remain multi-hop and the energy consumption will be lower. This is supported by the fact that long-range single hop communication consumes more energy than multi-hop [32]. Following this process the system reaches the next stage of the protocol *i.e.*, packet analysis at the BS.

### 4.6. BS Analysis

During the detection phase it is the role of the base station to analyse the received CPs and identify which node is misbehaving. This is done through a two pass analysis over an array containing the CPs. Since the analysis uses CPs of the other nodes to mark a particular node, the analysis of CPs received from the suspected malicious nodes are skipped (marked by “X”, Table 1 and Table 2). This is done to decrease the false positives and the false negatives. The values marked with “-” indicate no decision either due to a neighbouring malicious node or due to the position of that particular node in the network. The resulting “flags-array” with bit set to zero indicates the malicious node(s).

**Table 1:** Flags Bit Array—Pass I.

	S	S + 1	S + 2	S + 3	S + 4	BS
P1	1	1	1	0	- ^2^	1
PM	9	9	5	X ^1^	- ^2^	4

^1^ Analysis of CP received from suspected malicious node is skipped; ^2^ No decision.

**Table 2:** Flags Bit Array—Pass II.

	BS	S + 4	S + 3	S + 2	S + 1	S
P2	1	1	0	- ^2^	1	1
AM	5	8	X ^1^	8	- ^2^	- ^2^

^1^ Analysis of CP received from suspected malicious node is skipped, ^2^ No decision.

**Figure 12:**
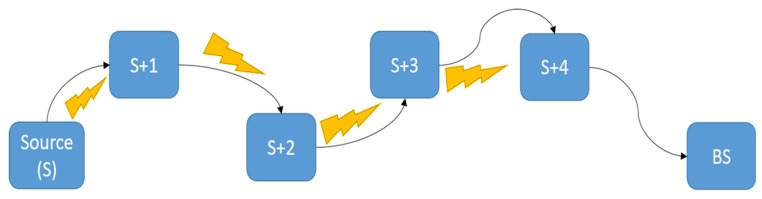
Testbed layout. The arrows represent DATA packets, and the bolts represent monitoring the neighbour node’s DATA sending habit.

Figure 12 shows the first pass, which consists of the BS verifying the neighbour DATA packets monitored by each node. Following is an example for the analysis of CPs:

1. Threshold is set to the expected number of packets at the BS, for a particular timeframe.
2. Since the BS and source (S) are trusted (Section 4.4), the flags-array bit for these nodes are set to 1 (see row P1, Table 2).
3. **If** the DATA count, monitored by node **j** (where j ϵ [S, S + 3]) is within the threshold range (*i.e.*, *DATA count − Threshold < Expected no. of packet drops*) **then** set flag for node j + 1 equal to 1, **Else** equal to 0.

Table 1 shows a sample first pass for the case when node S + 3 is malicious. Here P1 refers to Pass 1 and PM refers to packet monitoring of the neighbour node *i.e.*, no. of DATA packets forwarded by the neighbour node, j + 1.

This process will set all or some flags depending upon single or collaborative SF for nodes S + 1 to S + 4. Furthermore, the second pass is used to verify and/or fill the flags missed by the first pass.

In the second pass every node that has its control packets being analysed is not being judged simultaneously. This node may be judged only by the CPs of other nodes. The pass goes from BS side to source side as follows:

1. Threshold is set to the number of packets received at the BS (say 4, see row PM of BS in Table 1).
2. **If** the ACK count Monitored (AM) by node S+4 is within the threshold range (Table 2) **then** Set flag for S + 3 to 1.
3. **Else** **if** the same ACK count is not within the threshold range and
  a. **If** the DATA count monitored by node S+2 (Table 1) is out of range with respect to the no. of ACK packets monitored by BS (Table 2) *i.e.*, node S + 2 is lying about the no. of packets forwarded by S + 3, **then**
    i. Set flags for S + 2 and S + 3 to 0
    ii. Adjust the Threshold
  b. **Else if** the ACK count monitored by S + 4, is not within the range of the DATA count monitored by node S + 2 then
    i. Set flag for S + 3 to 0.
    ii. Adjust the Threshold

Finally, the malicious node(s) can now be identified by the corresponding zeroes in the flags bit array, see Table 2.

## 5. Details on Protocol Modifications

**Figure 13:**
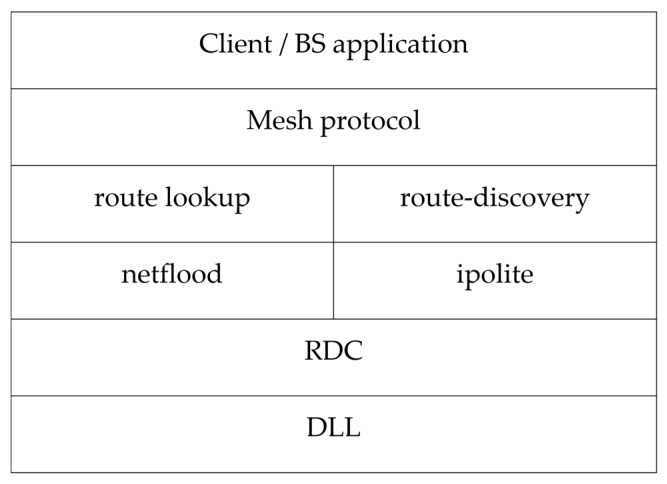
Network Stack.

The functioning of the mechanism in Section 4 is facilitated by certain modifications to the underlying protocols of the Contiki OS as shown in Figure 13. The lowest layer *i.e.*, the radio duty cycling layer (RDC), which is on top of the data link layer (DLL) [23] has been modified to incorporate the neighbour monitoring process of the system from Figure 8.

In the RDC layer the neighbour addresses are added to the 802.15.4 ACK, Figure 9b. This eases the job of nodes in capturing the correct ACK packet. In addition, the ACK capture and DATA capture functions are added to the code. These are responsible for managing the captured ACK and DATA packets by keeping separate associated counters. In this fashion the node has the knowledge of the number of packets sent and/or received by its neighbour(s).

Moving on to the mid-layers, the route-discovery is responsible for setting up a dynamic route between source and destination. This layer is modified to reverse the roles of RREQ and RREP packets and to include the hashing scheme (from Section 4.2). The hash in this case is calculated by upper layers and verified by this layer. If this verification results in a positive outcome a RREP packet is send back to the BS. Finally, this layer is responsible for tallying the received route packets against the malicious node list. If a RREQ or RREP packet is received from one of these malicious nodes the layer drops the packet and awaits a packet from one of the redundant nodes.

Next is the mesh protocol layer. This layer provides its own forwarding mechanism built on top of the route-discovery layer. It includes methods to send periodic data, forward data, and receive data. In addition, this layer monitors route changes and/or breaks in route and, if detected, the BS re-initiates route discovery.

## 6. Results

In order to better understand the protocol, its functionality using different metrics will be presented. The first metric is power/energy consumption. This particular metric was measured for two different scenarios: (1) Section 6.1 shows the Tmote Sky nodes on Contiki’s Cooja Simulator; and (2) Section 6.2 shows OpenMote nodes running the Contiki OS in real-time *i.e.*, the nodes are active and positioned in the university’s hallway. This is done to emulate a clinical/home patient monitoring system environment.

The second metric is network latency. This is shown in Section 6.3. The metric is explained for both single SF and collaborative SF attacks. Finally, the third metric analysed is the accuracy of the defence mechanism developed here. Here the metric is examined with the help of both Tmote Sky simulation (Cooja) and OpenMote real-time analysis and implementation.

During the analysis/experimental work for the above mentioned metrics; the system takes into account interference from other devices or technologies. In the case where Tmote Sky simulation (Cooja simulator) is used, the transmission range is set to 50 m and the interference range to 100 m. While the real-time testbed deployment of OpenMote has variable transmission and interference range. This is because of factors like indoor environment, temperature variations, node placement, antenna, other ISM devices, and people passing by. However, the programmable output power was configured to range between −24 dBm to 0 dBm, which means the maximum transmission range, in this case, is 1 m.

### 6.1. Power Consumption: Tmote Sky (Cooja)

In the first scenario a network consisting of Tmote sky (MSP430 + CC2420) nodes on the cooja simulator for Contiki OS is established. This setup builds around a unit disk graph medium (UDGM): *i.e.*, distance loss radio propagation model. This model includes interference with the capability of transmitting (TX) and receiving (RX) the packets with a success ratio probability [33].

Here, the energy consumption and power consumption of the modified routing protocol is determined in order to get a better understanding of the system, its lifetime and its availability. This is because the energy efficiency of routing protocols affects the system lifetime and system availability. The powertrace-profiler [34] is used which helps to build power consumption graphs for each node. Then, the energy consumption is derived from these graphs and compared against the system in [16]. The power consumed by the source node during two phases of the protocol *i.e.*, normal phase and detection phase is provided, where the former refers to the operation of the routing protocol without any interference from malicious node(s), and the latter refers to:

- process of malicious node detection at BS
- propagation of this information to the access points
- gathering and analysis of CPs at the BS, and
- propagation of the malicious node list.

**Figure 14:**
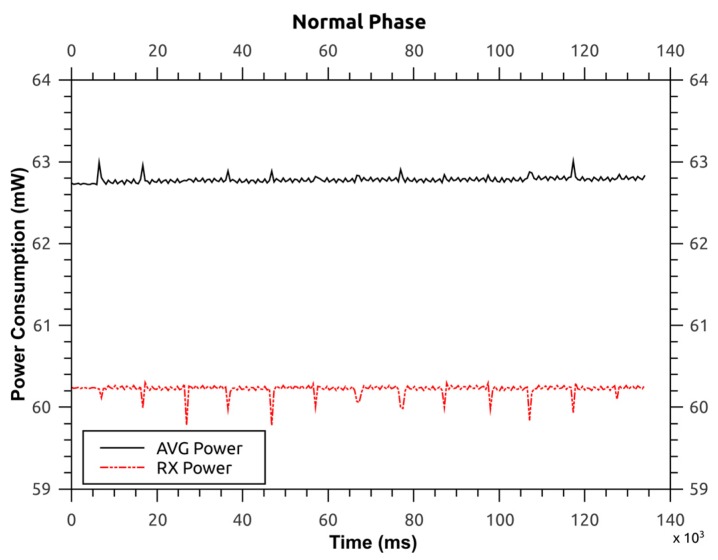
Power consumption for protocol—normal operation.

**Figure 15:**
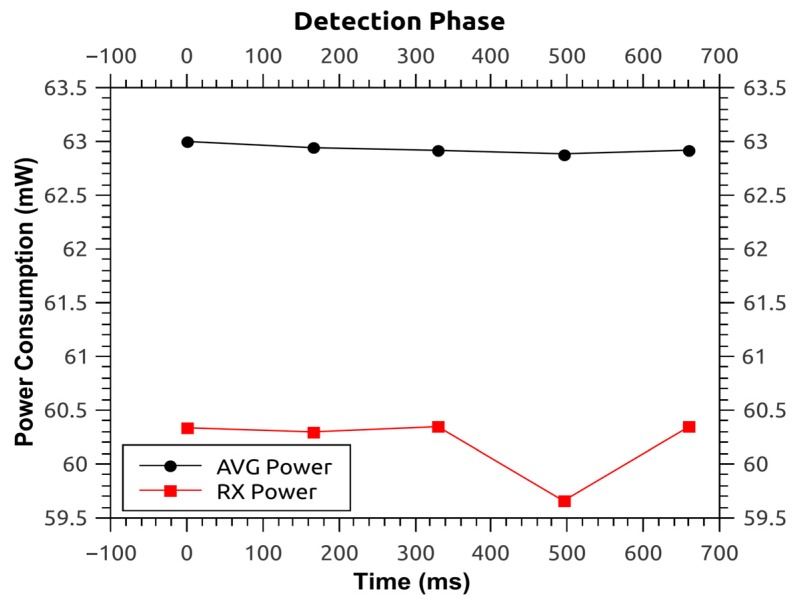
Power consumption for protocol—detection phase.

These phases have different power consumption as shown in Figure 14 and Figure 15. The former illustrates the average power during the normal phase of the protocol *i.e.*, 62.780 mW, and the latter illustrates the same for the detection phase of the protocol *i.e.*, 62.932 mW. As it can be seen there is a difference of only 152 μW, *i.e.*, 0.24%. So, it can be inferred that the power consumption during the detection phase is almost the same as the power consumption during the normal phase. This is because the protocol analysis has been shifted from the access points to the base station, thus, relieving the access points from unnecessary processing overhead and subsequent power consumption.

Comparing this work with the original protocol that is built-on Contiki OS, the latter has an average power consumption of 62.639 mW for the source node. This means that the modified protocol presented here consumes an extra 141 μW (0.22%) for normal mode and 293 μW (0.46%) for detection mode.

Furthermore, the time spent by the source node in normal and detection phases are 135 s and 665 ms, respectively (see Figure 14 and Figure 15). Thus the average energy consumption (given in Joules as this is the unit used in [16]) is 8.5 J and 41.8 mJ for normal and detection phase, respectively. Here, the detection phase consumes 8.46 J less than the normal phase. The energy consumption of the detection phase is lower than the normal phase because the duration of detection phase (0.665 s) is lower than that of the normal phase (135 s). Thus, the modified routing protocol has an advantage over the system in [16] because the latter consumes approximately 200 J (30%–33%) of energy more in its detection phase than its normal phase.

In addition, the average energy consumption between the two phases (8.5 J and 41.8 mJ) is 4.3 J, which is 30.7% lower than the average energy consumption of the system in [35] *i.e.*, 6.2 J.

### 6.2. Current Consumption: OpenMote

**Figure 16:**
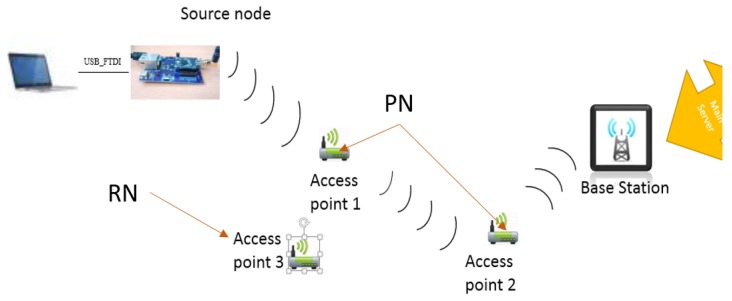
Experimental setup of the network.

In this section the real-time testbed implementation is analysed. This makes use of motes distributed through one of the hallways of our university, similar to the positions represented in Figure 16. This is done to emulate the home/clinic network environment.

This network consists of two types of connections *i.e.*, wired and wireless. Wired connection between the source node and laptop is provided through an OpenBase board [5]. This board connects the motes to the laptop using a serial port that is interfaced through a FTDI FT232R chip. The wireless network operates at 2.4 GHz and is compatible with the IEEE 802.15.4 standard.

The basic process is that the source node (ID: 4) is connected to a laptop for monitoring power consumption and the other nodes follow the order as shown in Figure 16. The source node sends patient data periodically towards the BS and the access points act as routers. Access point 3 is an observer *i.e.*, it does not take part in the data sending process but acts as a redundant node for the detection phase of the routing protocol. At the same time the whole network is being monitored on an individual level by each node with timing intervals that check whether the neighbours forward or drop any packets. At the end of this process any malicious node should be detected, if any, and the route corrected.

**Figure 17:**
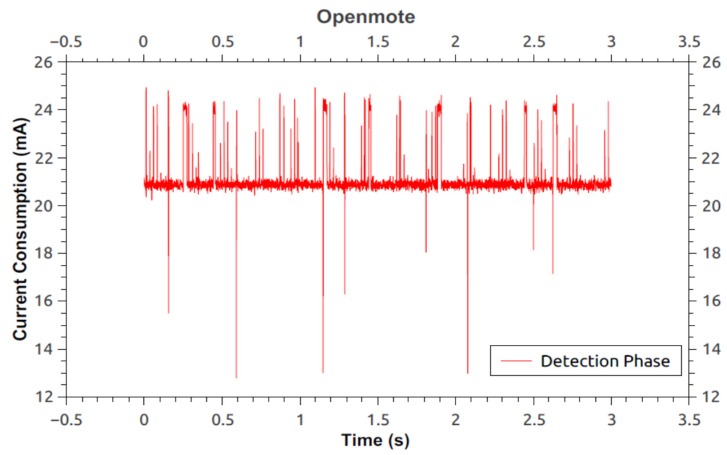
Current consumption of OpenMote running modified protocol during detection phase—nullrdc.

**Figure 18:**
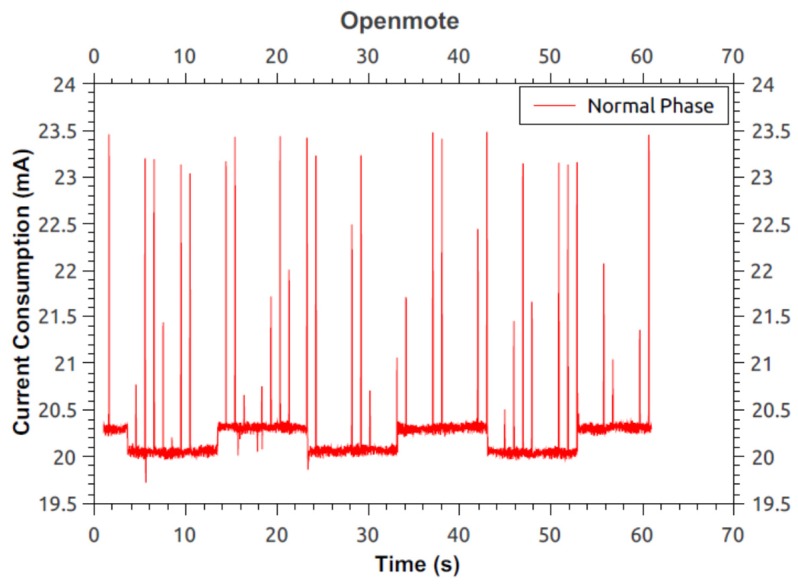
Current consumption of OpenMote running modified protocol during normal phase.

Following are the measurements taken for the two phases, *i.e.*, normal phase and detection phase. Here, the current was measured using the Agilent 66321D mobile communications DC source [36] and the 14565B device characterization software [37] with a sampling rate of 1364 Hz. Figure 17 shows the current consumption for the detection phase on an OpenMote. Measurement starts as soon as the LED lights up and finishes just before the LED turns off, *i.e.*, end of detection phase. The measurement provides the average current to be 21.0579 mA. Figure 18 illustrates the current consumption for the normal phase-on of the OpenMote. Here, the average current consumed by the source node (inclusive of all processes/components) is 20.223 mA.

From Figure 17 and Figure 18 it can be seen that there is an overhead of 834.9 μA during the detection phase of the protocol *i.e.*, an increase of 4.12%.

Considering this overhead the worst case scenario calculations can be made. This scenario refers to the detection phase being invoked after every ten data packets received at the base station. In this case a battery of 1100 mAh will last approximately 49 h; considering an excess of 10% to avoid the battery from going into deep discharge.

**Figure 19:**
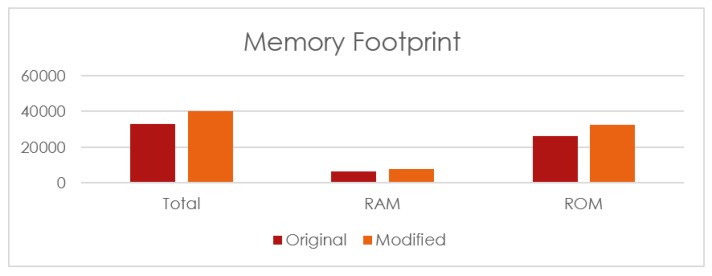
Memory footprint for the program on an OpenMote.

In addition to the current consumption the memory footprint is also shown in Figure 19. Here the original protocol consumes 32,815 bytes, *i.e.*, 6471 bytes of RAM and 26,344 bytes of ROM on the OpenMote platform. The modified routing protocol consumes a total of 40,322 bytes, *i.e.*, 7718 bytes of RAM and 32,604 bytes of ROM on the OpenMote. This amounts to an excess of 7507 bytes, *i.e.*, 22.8% increase for the modified protocol when compared to the original protocol.

The advantage provided by the modifications proposed here is that extra security is provided with low overhead with regards to energy/power and current consumption. However, the disadvantage is that the addition of security takes up to 7.5 kB more memory than the original protocol.

### 6.3. Latency: Network

In this section latency in the routing path is discussed. This latency is calculated by subtracting the time at which a particular node receives the detection information and the time at which the same node receives the ID of the malicious node.

This latency is illustrated for both single and collaborative selective forwarding attacks, Table 3 and Table 4 respectively. The main columns are described as follows:

1. “No. of nodes”: here the numbers with asterix are malicious nodes. The arrows show the data flow and “PN” refers to nodes on the routing path while “RN” refers to redundant nodes used for future new paths, Figure 16.
2. “Detection at”: shows the time (ms) at which SF detection information reaches a particular node.
3. “Mal info received”: shows the time (ms) at which the malicious node list reaches the same node.
4. “Latency”: gives the latency between the above two mentioned processes.

The following observations were noted:

1. From the tables it is seen that nodes closer to the BS update information more quickly compared to nodes farther away.
2. For the worst case scenario the maximum latency for Single SF detection and correction was 4767 ms.
3. When the malicious node drops control packets the latency is higher (19,650 ms). This is because the intermediate nodes must use netflood in-order to deliver the control packets to their destination (Section 4.5).
4. Finally, for collaborative SF, malicious nodes are detected, maximum two at a time.

### 6.4. Accuracy

The accuracy of the system in relation to the selective forwarding defence mechanism is discussed here. The accuracy is classified into two categories *i.e.*, detection and correction. The former refers to the ability of the system to accurately detect the malicious node(s), and the latter refers to the ability of the system in forming a malicious node free path.

The accuracy is calculated by examining the network layouts in Table A1, Table A2 and Table A3 (see Appendix A). Table A2 is dedicated to the ContikiMAC RDC mode, *i.e.*, the radio is being duty cycled for the system. In the tables the first column shows the data path and the asterix numbers refer to the malicious node ID(s). The second column shows the new path formed. The third column notifies whether detection was successful or not and reports false alarms. Finally, the fourth column shows the number of attempts it took to form a new path. Here, the redundant node (RN) takes the place of the malicious node and is shown in bold formatting.

The implementation setup is as follows: a certain node *i* is configured to drop packets selectively. It may or may not lie about the control packet information it generates. Additionally, for the collaborative case, node *i* − 1 is configured to lie about the number of data packets send by node *i*. This is done to cover up the malicious intent of node *i.*

Following this setup, the protocol was analysed for 29 different scenarios out of which four scenarios were implemented on the OpenMote hardware/testbed due to node availability and location limitations. The rest were implemented on the Cooja simulator for the Tmote Sky platform. The results from the simulation closely match the ones from the real-world deployment. This includes identifying the correct malicious node ID(s), false positives or negatives, and the formation of a new routing path (see Appendix A).

**Table 3:** Single Selective Forwarding.

Type	No. of Nodes	Node ID	Detection at (ms) (D)	Mal Info Received (ms) (M)	Latency (ms) M − D
Single SF	**5PN + 2RN** Orig: 4 → 6 → 5 → 3* → 2 → 1 New: 4 → 6 → 8 → 7 → 2 → 1	4	722,223	725,201	2978
		6	722,268	724,013	1745
		5	722,311	723,345	1034
		2	722,399	722,506	107
Single SF	**6PN + 1RN** Orig: 4 → 6 → 9 → 5 → 3* → 2 → 1 New: 4 → 6 → 9 → 5 → 7 → 2 → 1	4	742,919	747,686	4767
		6	742,970	746,498	3528
		9	743,021	745,795	2774
		5	743,071	745,127	2056
		2	743,173	743,295	122
Single SF^1^	**5PN + 1RN** Orig: 4 → 6 → 5 → 3* → 2 → 1 New: 4 → 6 → 8 → 7 → 2 → 1	4	719,981	739,631	19,650
		6	720,026	738,591	18,565
		5	720,069	737,921	17,852
		2	736,383 (with netflood)	736,482	99

* Malicious Node; ^1^ Malicious node drops control packets.

**Table 4:** Collaborative Selective Forwarding.

Type	No. of Nodes	Node ID	Detection at (ms) (D)	Mal Info Received (ms) (M)	Latency (ms) M − D
Collaborative SF	**5PN + 2RN** Orig: 4 → 6 → 5* → 3* → 2 → 1 New: 4 → 6 → 8 → 7 → 2 → 1	4	734,278	737,233	2955
		6	734,323	737,057	2734
		2	734,454	734,561	107
Collaborative SF Round 1	**5PN + 4RN** Orig: 4 → 6* → 5* → 3* → 2 → 1 New: 4 → 6* → 8* → 7 → 2 → 1	4	698,901	703,183	4282
		2	699,077	699,184	107
Round 2	Orig: 4 → 6* → 8* → 7 → 2 → 1 New: 4 → 9 → 10 → 7 → 2 → 1	4	1,710,799	1,712,192	1393
		7	1,710,931	1,711,184	253
		2	1,710,975	1,711,082	107

* Malicious Node.

The scenarios include single SF, collaborative SF, different number of nodes, and different malicious node IDs. As shown the protocol detects a selective forwarding attack with 96.5% accuracy and forms a malicious-node free path with 83% accuracy. In addition the protocol had 0.03% of false positives and 0.06% of false negatives only. The detection accuracy is higher compared to the systems in [38,39,40] where the same accuracy rates are 95% (when packet loss rate is 10%), 85%, and a little over 90%, respectively.

This increases confidence in the future use of this protocol in medical systems as it has a high rate of accuracy, low false alarm rate, and does not consume much additional power/energy to facilitate the inclusion of security. Of course, this protocol can also be used in other IoT situations that are susceptible to SF and black hole attacks.

## 7. Security Analysis

The inclusion of security is a vital parameter in the construction of a medical WSN. It is important to guarantee patient’s safety in addition to safeguarding the privacy of their data. Here the security of the proposed medical WSN system and the features it offers are discussed. This is an important step in the construction of the system as it helps in identifying any potential weaknesses in the systems security. The following analysis is based on the CIA (Confidentiality, Integrity, and Availability) triad:

Confidentiality: is provided through the use of a 256 bit key symmetric encryption (AES 256). This level of encryption and key size ensures that patient data remains confidential throughout the network.

Integrity: is provided for both control and data packets because the system makes use of a hash-based approach, which is also used to help prevent black hole attacks.

Availability: of service is ensured as the system is fortified against route-breaks, route-changes, replay attacks, and Denial of Service attacks. Thus ensuring availability of data at all times.

## 8. Conclusions

The healthcare industry requires the technological advances in WSNs and IoT applications to provide for the future demands and needs of patients and medical staff. These demands may be fulfilled by most IoT medical-based systems, but it is important to note that most of the systems currently on offer do not provide sufficient data security. The security of data routing has been seen as an unnecessary overhead in most of the published works in this field. As mentioned before, the DoS attack on data routing can have a significant impact on a medical-based WSN systems. In this paper an approach was presented to provide a scheme which prevented black hole attacks, and enabled the detection and correction of selective forwarding attacks.

The defence mechanism used against Selective forwarding attack provides a suitable way to deal with both single and collaborative attacks. This is useful because real-world attacks are often collaborative by nature between malicious nodes.

The detection rate of the proposed SF prevention system was found to be over 96%, which is an improvement over the accuracy of the system presented in [16]. While the correction rate was found to be 83% as mentioned in Section 6. The system examined took into consideration radio interference which is an added advantage for real-world implementations. This is because the system presented in this work was also implemented in a real-time testbed deployment using the OpenMote platform. This deployment helps to demonstrate the dependability and reliability of our system as simulations alone cannot capture the dynamics and complexity of the real-world.

The presented system provides several features that are useful in the real-world implementations of WSN in healthcare industries. For example, low energy consumption, high accuracy, confidentiality, integrity, and availability. These are important as they facilitate risk assessment and management of the system in addition to providing data and routing security.

### Future Work

In the future it is intended to expand the security of the system by introducing elliptic curve cryptography for encryption/authentication purposes in addition to the AES cipher that is used currently. The effect of excessive noise on the system will also be investigated to get a perspective of how a busy hospital environment can be controlled in contrast to the quieter (signal- and interference-wise) home setup.

## Supplementary Materials

The following are available online at http://www.mdpi.com/1424-8220/16/1/118/s1. Video S1. files are provided for illustrating the selective forwarding detection and correction mechanism as mentioned in Section 4.4.

## Appendix A

**Table A1:** Accuraccy check for single SF—nullrdc.

Original Path	New Path	Detection	Observations
**Tmote Sky**			
4, 2, 3*, 1	4, 2, 5, 1	Yes	1 attempt
4, 3*, 7, 6, 2, 5, 1	4, 8, 7, 6, 2, 5, 1	Yes	1 attempt
4, 8, 7, 3*, 5, 6, 2, 9, 1	4, 8, 7, 10, 5, 6, 2, 9, 1	Yes	2 attempts
4, 6, 3*, 8, 7, 5, 10, 2, 9, 1	4, 6, 12, 8, 7, 11, 10, 2, 9, 1	Yes & False Positive: ID 5	1 attempt
4, 10, 9, 5, 7, 8, 6, 2, 3*, 1	4, 10, 9, 5, 7, 8, 6, 2, 11, 1	Yes	2 attempts
**OpenMote**			
4, 3*, 6, 1	4, 2, 6, 1	Yes	1 attempt
4, 6, 3*, 1	X	No: False Negative	N/A
4, 3*, 2, 6, 1	X	Yes	N/A—Due to limitations on no of nodes

**A*** Represents the malicious node; **A** Represents the redundant node.

**Table A2:** Accuracy check for Single SF—ContikiMAC RDC.

Original Path	New Path	Detection	Observations
**Tmote Sky**			
4, 2, 3*, 7, 5, 1	4, 2, 8, 7, 5, 1	Yes	1 attempt:
4, 2, 3*, 7, 5, 8, 9, 10, 1	4, 2, 11, 7, 5, 8, 9, 10, 1	Yes	1 attempt:
4, 2, 7, 5, 3*, 9, 10, 1	X	Yes	N/A
4, 2, 5, 9, 8, 3*, 10, 1	4, 2, 5, 8, 8, 7, 10, 1	Yes	3 attempts:
4, 2, 5, 9, 8, 7, 3*, 10, 11, 1	4, 2, 5, 9, 8, 7, 12, 10, 11, 1	Yes	1 attempt
4, 2, 5, 9, 8, 7, 3*, 12, 10, 11, 1	4, 2, 5, 9, 8, 7, 13, 12, 10, 11, 1	Yes	4 attempts
4, 2, 5, 7, 8, 9, 3*, 12, 10, 11, 13, 1	4, 2, 5, 7, 8, 9, 14, 12, 10, 11, 13, 1	Yes	1 attempt

**A*** Represents the malicious node; **A** Represents the redundant node.

**Table A3:** Accuracy check for collaborative SF—nullrdc.

Original Path	New Path	Detection	Observations
**Tmote Sky**			
4, 6, **5***, **3***, 2, 1	4, 6, 8, 7, 2, 1	Yes	3 attempts
4, 6, 5, **7***, **3***, 2, 1	4, 6, 5, **9, 8**, 2, 1	Yes	1 attempt
4, 2, 7, 5, **8***, **3***, 6, 1	4, 2, 7, 5, **10, 9**, 6, 1	Yes	3 attempts
4, 6, **9***, **5***, 7, 3, 2, 8, 1	4, 6, **11**, **5^1^**, 7, 3, 2, 8, 1	Yes	1 attempt, 1 Error
4, 6, 11, **9***, **5*******, 2, 8, 7, 3, 1	4, 6, 11, **10**, **12**, 2, 8, 7, 3, 1	Yes	2 attempts
4, 7, 11, **9***, **3***, 2, 8, 6, 5, 1	4, 7, 11, **12, 13,** 2, 8, 6, 5, 1	Yes	3 attempts
4, 12, 11, **9*******, **3***, 1	4, 12, 11, **14**, **3** **^1^**, 1	Yes	1 attempt, 1 Error
4, 12, **9***, **3***, 11, 1	4, 12, **14**, **13**, 11, 1	Yes	3 attempts
4, 5, **6***, **2***, 3, 8, 11, 1	4, 5, **13**, **10**, 3, 8, 11, 1	Yes	2 attempts
4, 9, **6***, **2***, 10, 8, 1	4, 9, **11, 3**, 10, 8, 1	Yes	5 attempts
4, 2, 7, **9***, **5***, 3, 6, 1	4, 2, 7, **11, 10**, 3, 6, 1	Yes	3 attempts
(I) 4, 2, 10, **9***, **5***, 3, 6, 1	(I) 4, 2, 10, **7****^2^**, **8**, 3, 6, 1	(I) Yes: 5 & 9	1 attempt
		Mal node 7 infiltrates	
(II) 4, 2, 10, **7***, 8, 3, 6, 1	(II) 4, 2, 10, **11**, 8, 3, 6, 1	(II) Yes: 7	2 attempts
4, **6***, **2***, 8, 1	4, **9**, **10**, 8, 1	Yes	2 attempts
**OpenMote**			
4, **6***, **2***, 1	4, **8**, **10**, 1	Yes	1 attempt

**A*** Represents the malicious node; **A** Represents the redundant node; ^1^ Error node not identified as malicious; ^2^ Infiltration during the detection process.
